# Supplementary material for: Dynamic transcriptomic profiles of zebrafish gills in response to zinc supplementation
Source: BMC Genomics. 2010 Oct 11;11:553. doi: 10.1186/1471-2164-11-553 (PMC3091702; doi:10.1186/1471-2164-11-553)
Supplement: Additional file 2 — Interactive Direct Interaction Network representing the molecular interactions between zinc, copper, iron, calcium and proteins encoded by transcripts changed by zinc supplementation. Mini web-site containing index.html and hyperlinked pages in subdirectory describing a Direct Interaction Network automatically generated based on curated interactions contained within the proprietary PathwayArchitect database. Ovals represent proteins and the circles symbolize metal ions. Objects are coloured by their abundance in zebrafish at the time-point they were significantly different from the control is a scale from -4 fold (dark green) to +4 fold (dark red). Where significant differences were found at more than one time-point, the colour overlay shows expression at the first instance. Dark blue squares denote 'binding', and light blue squares 'expression'; green squares stand for 'regulation', green diamonds for 'metabolism', and green circles for 'promoter binding'. Arrow heads indicate directionality of the interaction where annotated. All nodes and edges can be further interrogated by selecting the relative area of the image. [file 1471-2164-11-553-S2.zip › PathwayArchitect Zn xs DIN/795374.html]

# REGULATION:

|  |  |
| --- | --- |
| Type | REGULATION |
| Effect | Positive |


---

|  |  |
| --- | --- |
| Score | 0 |


---

|  |  |
| --- | --- |
| Reference Count | 5 |


---

|  |  |
| --- | --- |
| Mechanism | Unknown |


---

|  |  |
| --- | --- |
| Reference:0 || Sentence | "These data indicate that under normal circumstances lung Cu,Zn-SOD and Mn-SOD are not affected by Cu or Mn deficiency." |
| PMID | 3406957 |
| Year | 1988 |
| Species | Mouse |
| Journal | Toxicol Lett |
| RefScore | 2 |
| Source | PArchNLP |
  |
|  |  |


---

|  |  |
| --- | --- |
 Reference:1 || Sentence | "Cu,Zn superoxide dismutase (SOD1) mutations cause one form of familial amyotrophic lateral sclerosis by a toxic gain of function that may be related to abnormal protein folding and aggregate formation." |
| PMID | 14622116 |
| Year | 2003 |
| Species | Mouse |
| Journal | J Neurochem |
| RefScore | 0 |
| Source | PArchNLP |
  ||  |  |


---

|  |  |
| --- | --- |
 Reference:2 || Sentence | "Ag-metallothionein (MT), which is associated with Ag, Cu, and Zn, and Cd-MT, which is associated with Cd, Cu, and Zn, were induced in the liver by the injection of Ag and Cd, respectively." |
| PMID | 6510399 |
| Year | 1984 |
| Species | Mouse |
| Journal | Environ Res |
| RefScore | 2 |
| Source | PArchNLP |
  ||  |  |


---

|  |  |
| --- | --- |
 Reference:3 || Sentence | Cu and Zn removal by the electrolyzed systems showed a complex response to increased ionic strength, which increased solid concentration, leading to lower Cu and Zn sorption. |
| PMID | 16009366 |
| Year | 2005 |
| Journal | J Colloid Interface Sci |
| RefScore | 3 |
| Source | PArchNLP |
  ||  |  |


---

|  |  |
| --- | --- |
 Reference:4 || Sentence | Cu-Zn catalyst (22.4% Cu and 26.4% Zn) was chemically treated with sulfuric acid at temperature 80 degrees C for 1 hr for the complete dissolving of copper and zinc into sulfate solution, then the produced solution was mixed with stoichiometric ratio of ferrous sulfate and the mixture was chemically precipitated as hydroxides followed by hydrothermal processing. |
| PMID | 16573080 |
| Year | 2006 |
| Journal | J Nanosci Nanotechnol |
| RefScore | 2 |
| Source | PArchNLP |
  |


---

|  |  |
| --- | --- |
